# A Hyperspectral Image Classification Framework with Spatial Pixel Pair Features

**DOI:** 10.3390/s17102421

**Published:** 2017-10-23

**Authors:** Lingyan Ran, Yanning Zhang, Wei Wei, Qilin Zhang

**Affiliations:** 1 School of Computer Science and Engineering, Northwestern Polytechnical University, Xi’an 710072, China; lingyanran@gmail.com; 2 Highly Automated Driving Team, HERE Technologies Automotive Division, Chicago, IL 60606, USA; samqzhang@gmail.com

**Keywords:** hyperspectral image classification, convolutional neural networks, spatial pixel pair features

## Abstract

During recent years, convolutional neural network (CNN)-based methods have been widely applied to hyperspectral image (HSI) classification by mostly mining the spectral variabilities. However, the spatial consistency in HSI is rarely discussed except as an extra convolutional channel. Very recently, the development of pixel pair features (PPF) for HSI classification offers a new way of incorporating spatial information. In this paper, we first propose an improved PPF-style feature, the spatial pixel pair feature (SPPF), that better exploits both the spatial/contextual information and spectral information. On top of the new SPPF, we further propose a flexible multi-stream CNN-based classification framework that is compatible with multiple in-stream sub-network designs. The proposed SPPF is different from the original PPF in its paring pixel selection strategy: only pixels immediately adjacent to the central one are eligible, therefore imposing stronger spatial regularization. Additionally, with off-the-shelf classification sub-network designs, the proposed multi-stream, late-fusion CNN-based framework outperforms competing ones without requiring extensive network configuration tuning. Experimental results on three publicly available datasets demonstrate the performance of the proposed SPPF-based HSI classification framework.

## 1. Introduction

Hyperspectral image (HSI) classification deals with the problem of pixel-wise labeling of the hyperspectral spectrum, which has historically been a heavily studied, but not yet perfectly solved problem in remote sensing. With the recent development of hyperspectral remote capturing sensors, HSIs normally contain millions of pixels with hundreds of spectral wavelengths (channels). The HSI classification is intrinsically challenging. While more and more high-dimensional HSIs accumulate and are made public available, ground truth labels remain scarce, due to the immense manual efforts required to collect them. In addition, the generalization ability of neural networks is unsatisfactory if they are trained with insufficient labeled data, due to the curse of dimensionality [1].

In the early days, conventional feature extraction and classifier design was popular among HSI classification practitioners. Favuel et al. [2] provide a detailed review of recent advances in this area. Many varieties of conventional features have been applied, including raw spectral pixels, spectral pixel patches and their dimension reduced versions by methods such as principal component analysis [3], manifold learning [4], sparse coding [5] and latent space methods [6,7,8,9]. Likewise, various conventional classifiers have been applied, such as support vector machines [10], Markov random fields [11], decision trees [12], etc. In particular, some early approaches have already tried the incorporation of spatial information by extracting large homogeneous regions using majority voting [13], watershed [14] or hierarchical segmentation [15]. Those two-stage (Stage 1: feature extraction; Stage 2: classification.) or multistage (in addition to the feature extraction stage and classification stage, there could be additional pre-/post-processing stages) methods suffer from some limitations. Firstly, it is highly time consuming to choose the optimal variant of conventional features and optimal parameter values for different HSI datasets, due to their wildly different physical properties (such as the number of channels/wavelengths) and visual appearances. Secondly, conventional classifiers have recently been outperformed by deep neural networks, particularly the convolutional neural network (CNN)-based ones.

In the past few years, deep learning methods have become popular for image classification and labeling problems [16,17,18], as an end-to-end solution that simultaneously extracts features and classifies. Unlike image capturing with regular cameras with only red, green and blue channels, HSIs are generated by the accumulation of many spectrum bands [19,20,21], with each pixel typically containing hundreds of narrow bands/channels. Many deep learning-based methods have been adapted to address the HSI classification problem, such as CNN variants [22,23,24,25], autoencoders [26,27,28,29] and deep belief networks (DBN) [30,31]. Despite their promising performance, the scarcity of training labels remains a great challenge.

Recently, Li et al. [32] proposed the pixel pair features (PPF) to mitigate the problem of training label shortage. Unlike conventional spectral pixel-based or patch-based methods, the PPF is purely based on combined pairs of pixels from the entire training set. This pixel-pairing process is used both in the training and testing (label prediction for the target dataset) stages. During the training process, two pixels are randomly chosen from the entire labeled training set, and the label of each PPF pair is deduced by a subtle rule based on the existing labels of both pixels. For pairs with pixels of the same labeled class, the pair label is trivially assigned as the shared pixel label. The interesting case arises when two pixels in a pair are of different labeled classes (which is especially likely for multi-class problems; the likelihood increases as the number of classes increases): the pair is labeled as “extra”, an auxiliary class artificially introduced. This random combination of pixels significantly increases the number of labeled training instances (within a total of $N_{c}$ labeled pixels of class *c* in the training set, there could be Nc2=12Nc2−12Nc randomly sampled PPF pairs for the class *c*), alleviating the training sample shortage.

Despite its success, the PPF implementation [32] includes randomly sampled pairs across the entire training set, which incurs very larger intra-class variances and changes the overall training set statistical distribution. Due to the intrinsic properties of HSI imaging, pixels with the same ground truth class labels could appear differently and statistically distribute differently across channels/wavelengths, especially for pixels geographically far apart from each other. Additionally, the PPF implementation [32] includes a pair label prediction rule inconsistent with its training pair labeling rule. At the testing (label prediction) stage, the “extra” class is deliberately removed, forcing the classifier (a Softmax layer) to pick an essentially wrong label for such PPF pairs.

Inspired by [32], a revamped version of the HSI classification feature is proposed in this paper, which is termed the “spatial pixel pair feature” (SPPF). The core differences of the proposed SPPF and the prior PPF are the geographically co-located pixel selection rule and the pair label assignment rule. For each location of interest, SPPF always selects the central pixel (at the location of interest) and one from its immediate eight-neighbor, at both the training and testing stage. In addition, an SPPF pixel pair is always labeled with the central pixel label, regardless of the other neighboring pixel.

Although the smaller neighborhood size yields fewer folds of training sample increase (the proposed SPPF offers up to eight-folds of training sample increase), the small neighborhood substantially decreases the intra-class variances. Statistically speaking, geographically co-located SPPF pixels are much more likely to share similar channel/wavelength measurement distributions than their PPF counterparts. Furthermore, the simple SPPF pair label assignment rule eliminates the complicated and possibly erroneous handling of “extra” class labels.

Alongside the shortage of training samples, the deep neural network structural design for the classifier is another challenge, especially for high dimensional HSI data. In this paper, we further propose a flexible multi-stream CNN-based classification framework that is compatible with multiple in-stream sub-networks. This framework is composed of a series of sub-networks/streams, each of which independently process one SPPF pixel pair. The outputs from these sub-networks are fused (in the “late fusion” fashion) with one average pooling layer that leverages the class variance and a few-fully connected (FC) layers for final predictions.

The remainder of this paper is structured as follows. Section 2 provides a brief overview of related work on HSI classification with deep neural networks. Section 3 presents the new SPPF feature and the new classification framework. Subsequently, Section 4 provides detailed experimental settings and results, with some discussions on potential future work. Finally, conclusions are drawn in Section 5.

## 2. Related Work

HSI classification has long been a popular research topic in the field of remote sensing, and the primary challenge is the highly-limited training labels. Recent breakthroughs were primarily achieved by deep learning methods, especially CNN-based ones [22,32].

Hu et al. [22] propose a CNN that directly processes each single HSI pixel (i.e., raw HSI pixel as the feature) and exploits the spatial information by embedding spectrum bands in lower dimensions. For notational simplicity, this method name is abbreviated to “Pixel-$CNN_{1}$” in this paper (no official abbreviation is provided in [22]), as it is based on individual HSI pixels, and the CNN inputs are all the channels/wavelengths from one pixel (hence the subscript 1). The Pixel-$CNN_{1}$ method outperforms many previous conventional methods without resorting to any prior knowledge or feature engineering. However, noise remains an open issue and heavily affects the prediction quality. Largely due to the lack of spatial information, the discontinuity artifacts (especially the ‘salt-and-pepper’ noise patterns) are widespread.

More recent efforts [33,34,35] have been made to incorporate spatial consistency in HSI classification. Qian et al. [36] especially claim that spatial information is often more critical than spectral information in the HSI classification task. Slavkovikj et al. [37] incorporate both spatial and spectral information by proposing a CNN that process a HSI pixel patch, which is abbreviated as Patch-$CNN_{9}$ in this paper (in [37], a patch typically covers 3×3 pixels, i.e., the inputs of CNN covers nine pixels, hence the 9 subscript). The Patch-$CNN_{9}$ method learns structured spatial-spectral information directly from the HSI data, while CNN extracts a hierarchy of increasingly spatial features [38]. Later, Yu et al. [39] proposed a similar approach with carefully crafted network structures to process three-dimensional training data. Meanwhile, Shi et al. [40] further enhanced the spatial consistency by adopting a 3D recurrent neural network (RNN) following the CNN processing. Recently, Zhang et al. [41] proposed a dual stream CNN, with one CNN stream extracting the spectral feature (similar to Pixel-$CNN_{1}$ [22]) and the other extracting the spatial-spectral feature (similar to Patch-$CNN_{9}$ [37]). Subsequently, both features are flattened and concatenated, before feeding into a prediction module.

Despite the success of the aforementioned approaches, the shortage of available training samples remains a vital challenge, especially when neural networks go deeper and wider [42], due to more parameters in a larger scale network model. To alleviate this problem, many efforts have been made. The first natural effort is better data augmentation. Slavkovikj et al. [37] introduce additional artificial noises based on the HSI class-specific spectral distributions. Granted that there is the risk of migrating the original spectral distribution, a reasonable amount of additive noise could lead to better generalization performance. Another way of augmenting training samples is proposed in [32], where randomly sampled pixel pairs (i.e., the PPF feature) from the entire training dataset are used. The PPF feature exploits the similarity between pixels of the same labeled class and ensures enough labeled PPF training pairs for its classifier.

Another group of work addresses the limited training labels challenge by exploiting the statistical characters in HSI channels/wavelengths. Methods like multi-scale feature extraction [23] extract multi-scale features using autoencoders followed by classifiers training. However, these methods are based on a lower dimensional spectral subspace, which may omit valuable information. Alternatively, some efforts have been made to combine HSI classification with auxiliary tasks such as super-pixel segmentation [43]. The super-pixel segmentation provides strong local consistency for labels and can also serve as a post-processing procedure. Romero et al. [44] present a greedy layer-wise unsupervised pre-training for CNNs, which leads to both performance gains and improved computational efficiency.

Additionally, the vast variations in the statistical distributions of channels/wavelengths also draw much research attention. Slavkovikj et al. [45] propose an unsupervised sub-feature learning method in the spectral domain. This dictionary learning-based method greatly enhanced the hyperspectral feature representations. Zabalza et al. [46] extract features from a segmented spectral space with autoencoders. The slicing of the original features greatly reduces the complexity of network design and improves the efficiency of data abstraction. Very recently, Ran et al. [47] propose the band-sensitive network (BsNet) for feature extraction from correlated band groups, with each band group earning a respective classification confidence. The BsNet label prediction is based on all available band group classification confidences.

## 3. SPPF and Proposed Classification Framework

In this section, the classification problem of HSI data is first formulated mathematically, followed by the introductions of early HSI features (raw spectral pixel feature and spectral pixel patch feature). Subsequently, the new SPPF feature is proposed and compared against the prior PPF feature. Finally, a new multi-stream CNN-based classification framework is introduced.

Let $X\in R^{W\times H\times D}$ be an HSI dataset, with *W*, *H*, *D* being the width (i.e., the total number of longitude resolution), height (i.e., the total number of latitude resolution) and dimension (i.e., spectral channels/wavelengths), respectively. Suppose among the total number of W×D pixels, *N* of them are labeled ones in the training set T:

(1) $$T={\left\{x_{i},y_{i}\right\}}_{i=1}^{N},\mathrm{where}\;x_{i}\in R^{D},y_{i}\in K,i=1,\cdots ,N.$$

$x_{i}$ is a *D*-dimensional real-valued spectral pixel measurement; $y_{i}$ is its corresponding integer valued label; $K=\left\{1,\cdots ,K\right\}$ is the set of labeled classes, with *K* being the total number of classes.

The goal of HSI classification is to find a prediction function $f:x_{j}\mapsto K$ for unlabeled pixels $x_{j}\notin T$, given the training set $T={\left\{x_{i},y_{i}\right\}}_{i=1}^{N}$.

For methods with incorporated spatial information (e.g., [37]), the prediction function *f* involves more inputs than $x_{j}$ itself. Additionally, a set of neighboring pixels $N(x_{i})$ is also *f*’s inputs. Therefore, the predicted label ${\hat{y}}_{j}$ is,

(2) $${\hat{y}}_{j}=f\left(x_{j},N\left(x_{j}\right)\right),x_{j}\notin T.$$

For notational simplicity, define $S_{i}$ as a set containing both the query pixel $x_{i}$ and its neighboring pixels $N(x_{i})$,

(3) $$S_{i}=\left\{x_{i},N\left(x_{i}\right)\right\},$$

and let $L_{i}$ represent the label of set $S_{i}$. With different choices of neighborhoods $N(x_{i})$, both $S_{i}$ and $L_{i}$ change accordingly. In the following sections, a bracketed number in the superscript of $S_{i}$ and $L_{i}$ is used to distinguish such variations.

### 3.1. Raw Spectral Pixel Feature

**Figure 1:**
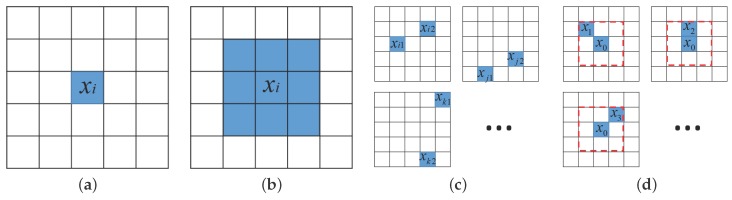
Illustrations of popular features used in HSI classification. Early CNN-based HSI classification methods are either based on raw spectral pixel feature [22] in (**a**) or spectral pixel patch feature [37] in (**b**). As shown in (**c**), [32] adopts a random sampling scheme across the entire training set to construct a large number of labeled pixel pair features (PPF) pairs. The proposed spatial pixel pair feature (SPPF) feature chooses a tight eight-neighbor as $N(x_{0})$, from which SPPF pairs are built, such as $\left\{x_{0},x_{1}\right\}$, $\left\{x_{0},x_{2}\right\}$, etc. (**a**) Raw spectral pixel feature; (**b**) spectral pixel patch feature; (**c**) PPF feature; (**d**) proposed SPPF feature.

As shown in Figure 1a, a spectral pixel is a basic component of HSI. The raw spectral pixel feature is used in early CNN-based work, such as Pixel-$CNN_{1}$ [22]. With such a feature, the label prediction task is:

(4) $$\begin{array}{c} {\hat{y}}_{j}^{\left(1\right)}=f^{\left(1\right)}\left(S_{j}^{\left(1\right)}\right),\mathrm{where}\;S_{j}^{\left(1\right)}=\left\{x_{j}\right\},\forall x_{j}\notin T. \end{array}$$

During $f^{\left(1\right)}$’s training process, labeling of $S_{i}^{\left(1\right)}$ is straightforward,

(5) $$L_{i}^{\left(1\right)}=y_{i},\forall x_{i}\in T,$$

where $y_{i}$ is the label associated with $x_{i}$.

Despite its simplicity, the lack of spatial consistency information often leads to erroneous predicted labels, especially within class boundaries.

### 3.2. Spectral Pixel Patch Feature

Proposed in [37], the spectral pixel patch feature uses the entire neighboring patch as basis for classification, as shown in Figure 1b,

(6) $${\hat{y}}_{j}^{\left(2\right)}=f^{\left(2\right)}\left(S_{j}^{\left(2\right)}\right),\mathrm{where}\;S_{j}^{\left(2\right)}=\left\{x_{j},N_{8}\left(x_{j}\right)\right\},\forall x_{j}\notin T,$$

where $N_{8}\left(x_{j}\right)$ denotes all eight pixels in $x_{j}$’s eight-neighbor.

During $f^{\left(2\right)}$’s training process, labeling of $S_{i}^{\left(2\right)}$ is completely based on the label $y_{i}$ of the central pixel $x_{i}$,

(7) $$L_{i}^{\left(2\right)}=y_{i},\forall x_{i}\in T,$$

In contrast to the raw spectral pixel feature, the spectral pixel patch feature provides spatial information and promotes local consistency in labeling, leading to smoother class regions. The introduction of spatial information in $N_{8}\left(x_{j}\right)$ significantly improves the prediction accuracy and contextual consistency.

### 3.3. PPF

The PPF feature [32] is illustrated in Figure 1c. Pairs of labeled pixels are randomly chosen across the entire training set T, and the label prediction is carried out by:

(8) $${\hat{y}}_{j}^{\left(3\right)}=f^{\left(3\right)}\left(S_{j}^{\left(3\right)}\right),\mathrm{where}\;S_{j}^{\left(3\right)}=\left\{x_{j},x_{q}\right\};x_{j},x_{q}\notin T,{\hat{y}}_{j}^{\left(3\right)}\in K.$$

During $f^{\left(3\right)}$’s training process, labeling of $S_{i}^{\left(3\right)}$ is based on the following rule,

(9) $$L_{i}^{\left(3\right)}=\left\{\begin{array}{cc} y_{i} & \mathrm{if}\;\;y_{i}=y_{p}\\ \Psi & \mathrm{if}\;\;y_{i}\neq y_{p} \end{array}\right.,\mathrm{where}\;x_{i},x_{p}\in T,$$

where $y_{i}$ and $y_{p}$ are the associated labels of $x_{i}$ and $x_{p}$ from pixel pair $\left\{x_{i},x_{p}\right\}$, respectively. Ψ is a newly introduced class label, denoting an “auxiliary” class not in the original K. In this paper, we set it as Ψ=K+1.

Comparing Equation (8) and Equation (9), it is evident that the spans of $L_{i}^{\left(3\right)}$ and ${\hat{y}}_{j}^{\left(3\right)}$ are different. The element Ψ in Equation (9) is outside the range of $f^{\left(3\right)}$. Therefore, there is a mismatch between the training labels and prediction labels, which could leads to suboptimal classification performance.

Additionally, there are no spatial constraints imposed on the choice of $x_{i}$ and $x_{p}$ while generating training samples ${\left\{S_{i}^{\left(3\right)},L_{i}^{\left(3\right)}\right\}}_{x_{i},x_{p}\in T}$ according to Equation (9). Therefore, $x_{i}$ and $x_{p}$ could be far apart from each other geographically. As Qian et al. argued in [36], spatial information could be more critical than its spectral counterpart, so such a pair generation scheme in Equation (8) could lead to high intra-class variances in training data $S_{i}^{\left(3\right)}$ and possibly confuses classifier $f^{\left(3\right)}$.

Practically, during the label prediction process in Equation (8), multiple $x_{q}$’s are normally chosen from the reference pixel $x_{j}$’s neighborhood $N(x_{j})$,

(10) $$M\left(j\right)=\mathrm{Card}\;\left(x_{p}|x_{p}\in N\left(x_{j}\right),x_{p}\notin T\right),\mathrm{where}\;x_{j}\notin T,$$

where M(j) denotes the total number of testing pixel pairs assembled for $x_{j}$. “Card” in Equation (10) represents the cardinality of a set. A series of $S_{j}^{\left(3\right)}$’s are constructed as:

(11) $$S_{j}^{\left(3\right)}\left(q_{1}\right)=\left\{x_{j},x_{q_{1}}\right\},\;S_{j}^{\left(3\right)}\left(q_{2}\right)=\left\{x_{j},x_{q_{2}}\right\},\cdots ,S_{j}^{\left(3\right)}\left(q_{M(j)}\right)=\left\{x_{j},x_{q_{M(j)}}\right\},$$

and their respective predictions are:

(12) $${\hat{y}}_{j}^{\left(3\right)}\left(1\right)=f^{\left(3\right)}\left(S_{j}^{\left(3\right)}\left(q_{1}\right)\right),\cdots ,{\hat{y}}_{j}^{\left(3\right)}\left(M\left(j\right)\right)=f^{\left(3\right)}\left(S_{j}^{\left(3\right)}\left(q_{M(j)}\right)\right).$$

The final predicted label is determined by a majority voting,

(13) $${\hat{y}}_{j}^{\left(3\right)}=\mathrm{mode}\;\left({\hat{y}}_{j}^{\left(3\right)}\left(1\right),\cdots ,{\hat{y}}_{j}^{\left(3\right)}\left(M\left(j\right)\right)\right),$$

where “mode” in Equation (13) denotes statistical mode (i.e., the value that appears most often).

### 3.4. Proposed SPPF

To address the lack of spatial constraints while selecting $\left\{x_{i},x_{p}\right\}$ and eliminating the extra Ψ label in Equation (9), the new SPPF feature is proposed and illustrated in Figure 1d. The label prediction is carried out by ${\hat{y}}_{j}^{\left(4\right)}\in K$,

(14) $${\hat{y}}_{j}^{\left(4\right)}=f^{\left(4\right)}\left({\left\{S_{j}^{\left(4\right)}\left(m\right)\right\}}_{m=1}^{8}\right),\;\mathrm{where}\;S_{j}^{\left(4\right)}\left(m\right)=\left\{x_{j},x_{q_{m}}\right\},\;x_{j}\notin T,{\left\{x_{q_{m}}\right\}}_{m=1}^{8}=N_{8}\left(x_{j}\right).$$

The SPPF prediction function $f^{\left(4\right)}$ always processes exactly eight sets of SPPF pairs ${\left\{S_{j}^{\left(4\right)}\left(m\right)\right\}}_{m=1}^{8}$, and these pairs holistically contribute to the prediction ${\hat{y}}_{j}^{\left(4\right)}$ without resorting to majority voting.

During $f^{\left(4\right)}$’s training process, only pixels from the reference pixel $x_{i}$’s eight neighbors are used for constructing $S_{i}^{\left(4\right)}\left(m\right)$. In addition, labeling of ${\left\{S_{i}^{\left(4\right)}\left(m\right)\right\}}_{m=1}^{8}$ is purely based on the central reference pixel $x_{i}$’s label $y_{i}$, eliminating any auxiliary class labels.

(15) $$L_{i}^{\left(4\right)}=y_{i},\forall x_{i}\in T,$$

The SPPF training set with pixel pairs and labels is:

(16) $${\left\{{\left\{S_{i}^{\left(4\right)}\left(m\right)\right\}}_{m=1}^{8},L_{i}^{\left(4\right)}\right\}}_{i=1}^{N},$$

where $L_{i}^{\left(4\right)}=y_{i}$, $S_{i}^{\left(4\right)}\left(m\right)=\left\{x_{i},x_{p_{m}}\right\},{\left\{x_{p_{m}}\right\}}_{m=1}^{8}=N_{8}\left(x_{i}\right),\forall x_{i}\in T$.

### 3.5. Proposed Classification Framework with SPPF

**Figure 2:**
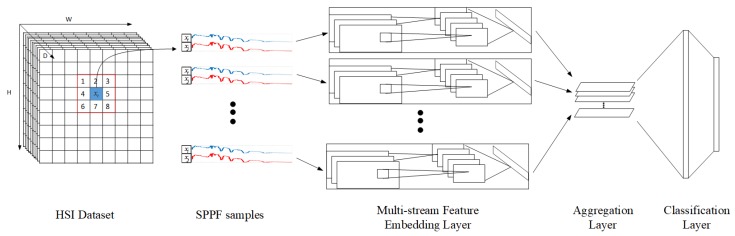
Proposed classification framework for HSI classification based on SPPF features.

On top of the proposed SPPF feature, a multi-stream CNN architecture is proposed for classification, as shown in Figure 2. Overall, there are three major component layers: firstly, the multi-stream feature embedding layers; secondly, an aggregation layer; and finally, a classification layer.

The multi-stream feature embedding layers are designed to extract discriminative features from SPPFs. These layers are grouped into eight streams/sub-networks, each of which processes a single $S_{i}^{\left(4\right)}\left(m\right)$, where m=1,⋯,8. A major advantage of this design is the flexibility of incorporating various nets as streams/sub-networks. We have adopted both classical CNN implementations, as well as alternatives such as [47]. All sub-networks have their input layers slightly adjusted to fit the pixel pair inputs; and last scoring layers (i.e., SoftMax layers) are removed. After passing the multi-stream feature embedding layers, HSI data are transformed into eight streams of *K*-dimensional feature vectors, ready to be fed to the next aggregation layer.

Empirically, the overall classification performance is insensitive to different choices of sub-networks, given a reasonable sub-network scale. In fact, due to the limited amount of training data, slightly shallower/simpler networks enjoy a marginal performance advantage.

The aggregation layer is one average pooling layer, which additively combines information from all streams together, leading to its robustness against noises and invariance from local rotations. The output of the prior multi-stream embedded layer is of dimension $F_{K\times 1}\left(m\right)$; m=1,⋯,8 are first concatenated to form F(c):

(17) $$F_{K\times 8}\left(c\right)={\left[F_{K\times 1}{\left(1\right)}^{T},\cdots ,F_{K\times 1}{\left(8\right)}^{T}\right]}^{T}.$$

Subsequently, an average pooling layer process F(c) outputs a vector $F_{K\times 1}\left(a\right)$. Even if there are noise contaminations in some streams/sub-networks, $F_{K\times 1}\left(a\right)$ is less susceptible to them, thanks to the averaging effect. Lastly, two fully-connected (FC) layers and a SoftMax layer serve as the classification layers, providing confidence scores and prediction labels.

## 4. Experimental Results and Discussion

In this section, we give the detailed configuration description of the datasets we use and the models we build for analyzing the new SPPF and the proposed classification framework. For HSI classification measurement, we choose the overall accuracy (OA) and average class accuracy (AA) as the evaluation strategy (the overall accuracy is defined as the ratio of correctly labeled samples to all test samples, and the average accuracy is calculated by simply averaging the accuracies for each class.).

### 4.1. Dataset Description

We test the proposed framework and competing state-of-the-art methods on three publicly available HSI datasets. All datasets are open accessible online (http://www.ehu.eus/ccwintco/index.php?title=Hyperspectral_Remote_Sensing_Scenes).

India Pines dataset: The NASA Airborne Visible/Infrared Imaging Spectrometer (AVIRIS) Indian Pines image was captured over the agricultural Indian Pine test site located in the northwest of Indiana. The spectral image has a spatial resolution of 145×145 and a range of 220 spectral bands from 0.38–2.5 μm. Prior to commencing the experiments, the water absorption bands were removed. Hence, we are dealing with a 200-band length spectrum. What is more, the labeled classes are highly unbalanced. We choose nine out of 16 classes, which consists of more than 400 samples. In total, 9234 samples are left for further analysis.

University of Pavia dataset: The University of Pavia image (PaviaU) was acquired by the ROSIS-03 sensor over the University of Pavia, Italy. The image measures 610×340 with a spatial resolution of 1.3 m per pixel. There are 115 channels whose coverage ranges from 0.43–0.86 μm, and 12 absorption bands were discarded for noise concern. There are nine different classes in the PaviaU reference map and 42,776 labeled samples used in this paper.

Salinas scene dataset: This scene is also collected by the 224-band AVIRIS sensor over Salinas Valley, California, and is characterized by high spatial resolution (512×217 3.7-m pixels). As with Indian Pines scene, we discarded the 20 water absorption bands, in this case bands with the numbers: [108–112], [154–167], 224. This image was available only as at-sensor radiance data. It includes vegetables, bare soils and vineyard fields. The Salinas ground-truth contains 16 classes and 54,129 samples.

### 4.2. Model Setup and Training

For efficiency demonstration of the proposed method, we first have some classical shallow classification methods built in the comparison experiment, for example SVM [10] and ELM [48]. We use the LIBSVM [49] and ELM (http://www.ntu.edu.sg/home/egbhuang/elm_codes.html) development toolkit for building those two models separately. For the SVM model, we use the polynomial kernel function, with γ equal to one and the rest of the hyper-parameters set as the default. For the ELM model, we set the number of hidden neurons as 7000 and choose the Sine function as the activation function. Predictions are given by each model regarding raw input spectrum pixels.

As for CNN-based deep models, we have reproduced three works, CNN from [37], BsNet [47] and PPF [32]. To distinguish, we have adjusted the model’s name in different case. For example, we have $CNN_{1}$ and $CNN_{9}$ to present the model in [37] working on the spectrum pixel (one pixel) and spatial patch (nine pixels), respectively. $CNN_{2}$ is for the model in [37] when it is embedded into the proposed framework working on a pixel pair (two pixels). We further simplified the $CNN_{2}$ subnetwork, which we quote as $CNN_{2}$-lite in this paper, with the number of neurons in the fully-connected layers set to 400 and 200, respectively. In this way, the total parameters have greatly dropped from 4,650,697 down to 2,100,297 for one single subnetwork. Meanwhile, the chance of overfitting is greatly decreased. In Table 1, we have listed out the different configurations for better clarification.

For the PPF framework [32], we have adjusted the voting size during the testing stage to be three (the default value is five), which matches the size with other spatial spectral methods ($CNN_{9}$) in our paper.

When building the proposed framework in Figure 2, we choose $CNN_{2}$, $CNN_{2}$-lite and BsNet as the subnetworks, which we refer to as SPPF framework ($CNN_{2}$), SPPF framework ($CNN_{2}$-lite), and SPPF framework (BsNet) respectively. The subnetworks are initialized with default configurations and further fine-tuned for advantageous performance during the training stage of the whole framework. The rest of the parameters are set to be the default.

The proposed and comparative CNN models are all developed using the Torch7 deep learning package [50], which makes many efforts to improve the performance and efficacy of benchmark deep learning methods. The training procedures for all models are run on an Intel Core-i7 3.4-GHz PC with an Nvidia Titan X GPU. For the proposed framework, we use the tricks in efficient backprop [51] for initialization and the adaptive subgradient online learning (Adagrad) strategy [52] for optimization, which allows us to derive strong regret guarantees. Further details and analysis of the performance of the network configurations are given in the following subsection of the experimental classification results.

### 4.3. Configuration Tests

For establishing a stable architecture, we have tested several configurations and verifications on different datasets. The major configurations are kept in the same, except parameters to be verified.

**Figure 3:**
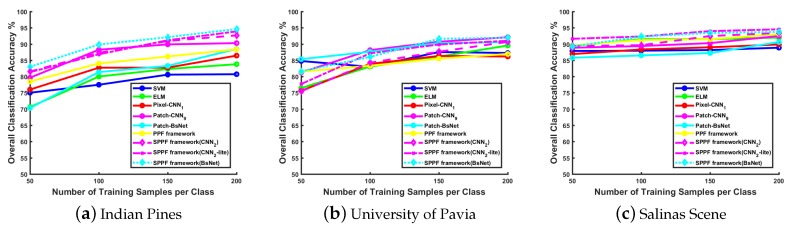
Stability comparison results on three datasets with increasing number of training samples. Generally, more training samples result in models having better performance.

Effect of training samples: Figure 3 illustrates the classification performance with various numbers of training samples on different datasets. Generally, when the training size increases, the performance of all methods has a noticeable improvement. In the following experiments, we choose 200 samples from each class, which gives a relatively larger number of training samples against the overfitting problem. The remaining instances of each class are grouped into the testing set. The detailed dataset class description and configuration are presented in Table 2. All those samples are normalized to a new range with zero mean and unit variance before they are fed into different models.

**Figure 4:**
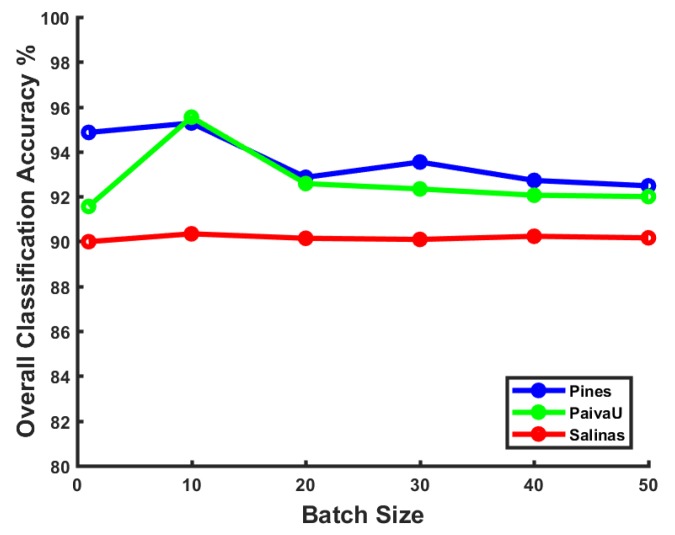
Batch size influence on training the SPPF framework ($CNN_{2}$-lite) with different datasets.

Effect of batch size: The size of sample batches during the training stage can also have some impact on the model performance, especially for optimization methods like stochastic gradient decent. We have tested various batch size values ranging from 1–50 on three datasets. The results are shown in Figure 4. We can figure out that a larger batch size does not necessarily always help the model obtain better performance. Although the mini-batch training trick has the strength of faster convergence maintenance, a larger batch size with limited samples disturbs the convergence stability occasionally. In the following experiments, we choose a batch of 10 samples for training, which gets the top classification precision in our framework.

Effect of the average pooling layer: The average pooling layer is designed to introduce the framework with noise and variance stability, which also shares a similar property as the voting stage in the PPF framework. As can be seen from Table 3, the precision improved with about 0.8% for the Pines and Salinas datasets. For the PaviaU dataset, the influence is not that obvious. As the spectrum in PaviaU is much shorter (103 bands in fact), noises may not be that essential as the remaining two dataset with over 200 bands. With no harm to the final precision, we would keep the averaging layer in the following experiments for all datasets.

Effect of FC layers: There is no doubt that the capacity of fully-connected layers can directly influence the final classification accuracy. While a larger size of FC layers increases the classifier capacity, models with smaller ones are easier to train and suit problems with limited training samples better. During the framework design, we have tried to use two and three fully-connected layers alternatively. As shown in Table 4, the model with $CNN_{2}$-lite as subnetworks working on different datasets shows various performance results. Take the Pines dataset as an example: when we change from two FC layers to three FC layers, the precision obtains about a 2% decrease. Meanwhile, we have a slight drop on the PaviaU dataset and about 0.5% improvement on the Salinas dataset. One reason might be that the higher capacity of a fully-connected classification module is needed when we have a superior number of classes in the dataset. In the remaining experiments, to make one unified framework, we choose two FC layers as the prediction module.

### 4.4. Classification Results

In this subsection, we give the detailed results from the proposed model, as well as comparative ones on three datasets. Table 8, Table 9 and Table 10 list out the details of OA and AA for the Pines, PaviaU and Salinas datasets, respectively.

One obvious observation would be that CNN-based deep models (Columns 4–10) show boosting performance over shallow models like SVM (Column 2) and ELM (Column 3). That reflects the booming development of deep learning methods in recent years with competing accuracy.

Spatial consistency reserves a strong constraint for HSI classification. For spatial spectral-based CNN models (Column 5), there is about a 4% improvement compared to spectral alone (Column 4). The introduction of neighbor pixels not only gives models more information during prediction, but also maintains contextual consistency.

For SPPF-based models, it is verified that the performance of conventional deep learning solutions for HSI classification can be further boosted by the proposed SPPF-based framework. When comparing Column 5 with Column 7 and Column 6 with Column 10, we can observe that both CNN [37] and BsNet get better performance when embedded in the proposed framework. This confirms that the proposed framework works efficiently without any increasing of training samples. Besides, the SPPF framework with $CNN_{2}$-lite (Column 9) shows even better results than $CNN_{2}$ (Column 8), which contains much more parameters than the previous one. Hence, the framework also shows an advantage in controlling the model size on the same task. Moreover, we do not need that many parameters to maintain a good performance if the problem has limited samples. To be concise, by simply changing from conventional CNN models to the SPPF framework, we can greatly shrink the model size and meanwhile relieve the over-fitting chance.

**Figure 5:**
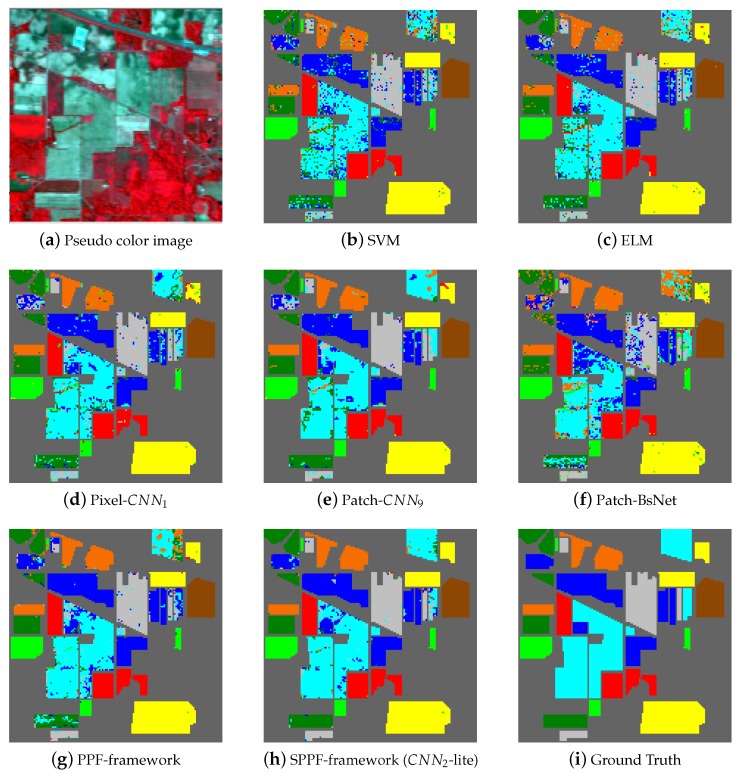
Results on the University of Pines dataset. (**a**) Pseudo color image of the scene. (**b–h**) Results from competing methods. (**i**) The ground truth. Our algorithm achieves the best classification accuracy among the competing methods. (Best viewed in color.)

**Figure 6:**
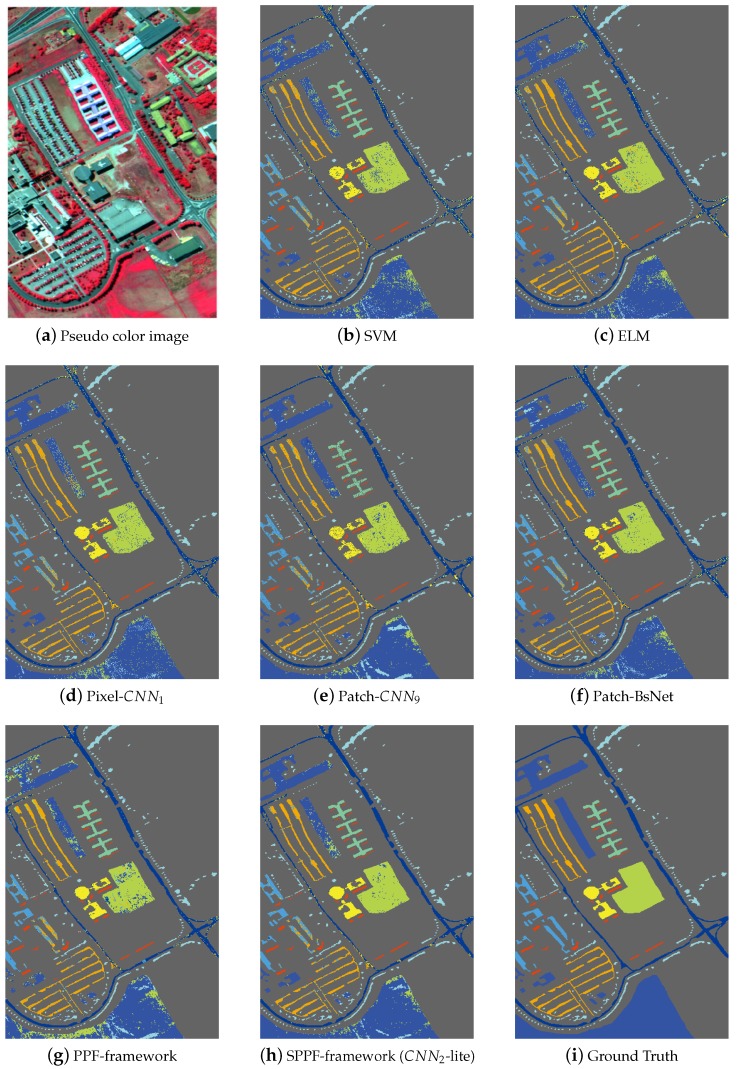
Results on the University of Pavia dataset. (**a**) Pseudo color image of the scene. (**b–h**) Results from competing methods. (**i**) The ground truth. Our algorithm achieves the best classification accuracy among the competing methods. (Best viewed in color.)

**Figure 7:**
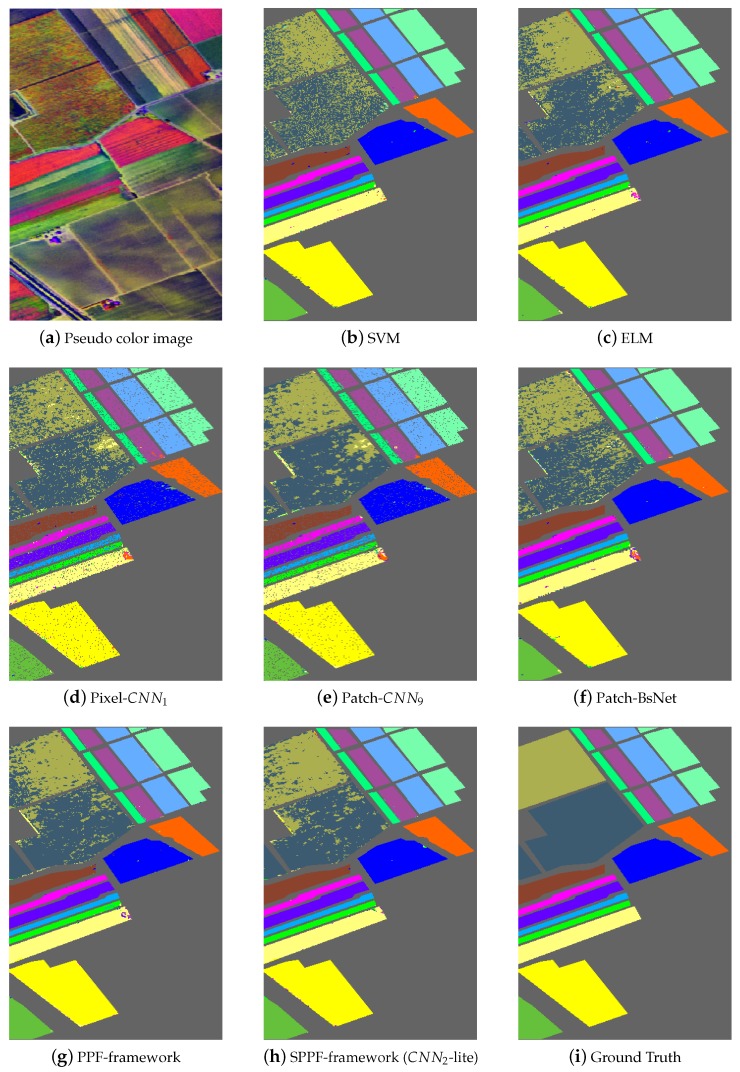
Results on the University of Salinas dataset. (**a**) Pseudo color image of the scene. (**b–h**) Results from competing methods. (**i**) The ground truth. Our algorithm achieves the best classification accuracy among the competing methods. (Best viewed in color.)

Figure 5, Figure 6 and Figure 7 shows the thematic maps accompanying the results from Table 8, Table 9 and Table 10 separately. In Figure 5a, we give one pseudo color image of the Indian Pines dataset for a better illustration of the natural scene and also for the declaration of why spatial consistency holds. Figure 5b–g shows the classification results from SVM, ELM, Pixel-$CNN_{1}$, Patch-$CNN_{9}$, BsNet and the PPF framework, Figure 5h shows the result from the SPPF framework ($CNN_{2}$-lite), which is the best one of the SPPF models. Lastly, Figure 5i gives the ground truth map. Similar results are also shown in Figure 6 and Figure 7 for the remaining two datasets. It is straightforward to tell that the results from SPPF show superior performance to the previous methods, where less noisy labels are given and the spatial consistency is improved.

The statistical differences of SPPF over competitive models with the standardized McNemar test are given in Table 5. According to the definition in [53], the value *Z* from McNemar’s test reflects that one method is significantly statistically different from another when its absolute value is larger than 2.58. The larger value of Z shows better accuracy improvement. In the table, all the values show that the proposed SPPF outperforms previous CNN-based solutions.

Table 6 gives the total time consumption of training models and the average time cost of testing one sample. All the experiments are fulfilled with the Torch7 package using the same PC described in Section 4.2. It is worth mentioning that as we have set the early-stopping criterion when training error stops dropping or validation accuracy starts decreasing, the training time does not necessarily reflect each model’s complexity. The testing time, on the other hand, is more suitable as a reflection of the model complexity. It is reasonable for the PPF model to take a much longer training time, as it is dealing with many more samples. Besides, the relatively high testing consumption of PPF relies on the fact that the model should be run eight times before the voting stage. Meanwhile, the voting stage, which is not a part of the model, also requires extra time. When conventional CNN models are embedded into the SPPF framework, their time consumptions increase accordingly. However, the testing time difference of BsNet when embedded is not that obvious. That is because most of the time consumption comes from the grouping stage, which shows no difference in those cases. Besides, the input channels are decreased, which also helps with time saving.

### 4.5. Discussion

Based on the comparison tests in Section 4.3 and the classification results in Section 4.4, a brief performance analysis is included in this section.

The primary comparative advantage of the proposed SPPF framework is the incorporation of contextual information, which has significantly boosted the spatial consistency. Unlike conventional CNN-based HSI classification methods (e.g., $CNN_{1}$, $CNN_{9}$), the discontinuity artifact (such mislabeled pixels distribute randomly and sparsely and form a salt-and-pepper noise pattern in Figure 5, Figure 6 and Figure 7) has greatly attenuated. In addition, the proposed spatial information preserving pixel pair selection scheme in Section 3.4 are also evidently proven to be superior to the original PPF in Table 8, Table 9 and Table 10.

The advantages of the subnetwork-based multi-stream framework are also verified. Firstly, this particular design offers a scalable network structure template without requiring the formidable manual selection process to determine a suitable sub-network configuration. According to the last three columns in Table 8, Table 9 and Table 10, this multi-stream framework is robust to different selections of subnetworks: both $CNN_{2}$ and BsNet provide decent performances, only marginally inferior to the optimal $CNN_{2}$-lite. Incidentally, from the comparison between $CNN_{2}$ and $CNN_{2}$-lite in the SPPF framework, it appears that more parameters (in $CNN_{2}$) do not necessarily guarantee improved classification accuracy.

For future work, the incorporation of pixel-group features with three or more pixel-combinations shows great potential. Being a natural extension to the pixel pair feature, the triplet pixel feature is introduced and briefly tested bellow, with initial performance already better than the original pixel pair feature. Further analysis of the choices of adjacent pixels and label assignment strategy is going to be included in our future work.

Effect of spatial triplet pixel features: Since we have designed the framework to adopt multiple pixel-pairs as input rather than the normal patch, it is interesting to explore if more pixels as features can work better. In this subsection, we use triplet pixels as an input feature for subnetworks. Comparing with pixel-pairs, triplet pixels show the subnetworks more information and also greater potential of better classification capacity. For achieving this experiment, the first convolutional layer in subnetwork is changed to three input channels, slightly different from the two input channels for SPPF framework, which we present as $CNN_{3}$-lite. The remainder of the whole framework is kept the same. For sample formatting, we have instance $S_{i}^{\left(5\right)}\left(t\right)=\left\{x_{i},x_{p},x_{l}\right\}$, with $x_{i}$ being the query central pixel, $x_{p},x_{l}\in N_{8}\left(x_{i}\right)$. The label prediction for one triplet pixel is carried out by ${\hat{y}}_{j}^{\left(5\right)}\in K$,

(18) $${\hat{y}}_{j}^{\left(5\right)}=f^{\left(5\right)}\left({\left\{S_{j}^{\left(5\right)}\left(t\right)\right\}}_{t=1}^{T}\right),\;\mathrm{where}\;S_{j}^{\left(5\right)}\left(t\right)=\left\{x_{j},x_{q_{t}},x_{r_{t}}\right\},\;x_{j}\notin T,x_{q_{t}},x_{r_{t}}\in N_{8}\left(x_{j}\right),\;t=1,\cdots ,T,$$

where T=83. The training label for this spatial triplet pixel features is defined as:

(19) $$L_{i}^{\left(5\right)}=y_{i},\forall x_{i}\in T.$$

Since the combination of triplets are various for a α-neighbor, in practice, we randomly choose eight triplets to fit into the framework presented previously. The final prediction results are shown in Table 7. The precision shows noticeable increase on all datasets. This observation embraces our proposal that using spatial consistency can greatly improve the models’ capacity.

## 5. Conclusions

In this paper, a new spatial pixel pair-based, multi-stream convolutional neural network is proposed for HSI classification applications. Unlike conventional neural networks that regard the spatial information purely as an extensional channel, the proposed framework captures additional spatial information via a series of subnetworks (streams) with flexible subnetwork configurations and achieves superior classification accuracy on three publicly available datasets. Further discussion on grouping pixels for better improvement is open for future work. Source codes are available on the project page: https://hijeffery.github.io/HSI-SPPF/.

## Figures and Tables

**Table 1:** Configuration list of selected models working on the India Pines dataset for the illustration of input and output modifications. FC, fully-connected.

	${\mathrm{CNN}}_{1}$	${\mathrm{CNN}}_{9}$	${\mathrm{CNN}}_{2}$	${\mathrm{CNN}}_{2}$-Lite
Input	1 × 200 × 1	9 × 200 × 1	2 × 200 × 1	2 × 200 × 1
Conv1	(1 × 16) #32	(9 × 16) #32	(2 × 16) #32	(2 × 16) #32
Conv2	(1 × 16) #32	(1 × 16) #32	(1 × 16) #32	(2 × 16) #32
Conv3	(1 × 16) #32	(1 × 16) #32	(1 × 16) #32	(2 × 16) #32
FC1	4960–800	4960–800	4960–800	4960–400
FC2	800–800	800–800	800–800	400–200
FC3	800-*K*	800-*K*	800-*K*	200-*K*
Output	SoftMax	SoftMax	-	-

**Table 2:** Detailed configuration of the three datasets (Pines, University of Pavia (PaviaU) and Salinas) used in this paper. For each class, 200 samples are randomly selected as the training set and the rest as the testing set. For Pines, we use only the top 9 classes with the largest number of samples.

No.	Pines			PaviaU			Salinas		
	Class Name	Train	Test	Class Name	Train	Test	Class Name	Train	Test
1	Corn-notill	200	1228	Asphalt	200	6431	Brocoli_1	200	1809
2	Corn-mintill	200	630	Meadows	200	18,449	Brocoli_2	200	3526
3	Grass-pasture	200	283	Gravel	200	1899	Fallow	200	1776
4	Grass-trees	200	530	Trees	200	2864	Fallow_plow	200	1194
5	Hay-win.	200	278	Sheets	200	1145	Fallow_smooth	200	2478
6	Soy.-notill	200	772	Bare Soil	200	4829	Stubble	200	3759
7	Soy.-mintill	200	2255	Bitumen	200	1130	Celery	200	3379
8	Soy.-clean	200	393	Bricks	200	3482	Grapes	200	11,071
9	Woods	200	1065	Shadows	200	747	Soil_vinyard	200	6003
10							Corn_weeds	200	3078
11							Lettuce_4wk	200	868
12							Lettuce_5wk	200	1727
13							Lettuce_6wk	200	716
14							Lettuce_7wk	200	870
15							Vinyard_un.	200	7068
16							Vinyard_ve.	200	1607
Sum		1800	7434		1800	40,976		3200	50,929

**Table 3:** Classification performance of the SPPF framework using $CNN_{2}$-lite as the subnetwork with/without the average pooling layer (OA shown in percentage).

	Pines	PaviaU	Salinas
with avg layer	**96.02**	**92.73**	**95.16**
without avg layer	95.33	92.70	94.84

**Table 4:** Classification performance of the SPPF framework using $CNN_{2}$-lite as subnetworks with different numbers of FC layers (OA shown in percentage).

	Pines	PaviaU	Salinas
2 FC layers	**93.89**	**91.02**	94.54
3 FC layers	91.79	90.73	**95.13**

**Table 5:** Statistical difference of SPPF over competitive models with the standardized McNemar test. BsNet, band-sensitive network.

	SPPF vs. ${\mathrm{CNN}}_{1}$	SPPF vs. ${\mathrm{CNN}}_{9}$	SPPF vs. BsNet	SPPF vs. PPF
Z	21.01	9.04	20.02	20.00

**Table 6:** Time consumption in training and testing deep models on the Pines dataset.

	${\mathrm{CNN}}_{1}$	${\mathrm{CNN}}_{9}$	BsNet	PPF	SPPF		
					${\mathrm{CNN}}_{2}$	${\mathrm{CNN}}_{2}$-Lite	BsNet
Training (h)	0.80	0.50	0.70	46.1	1.48	1.42	10.15
Testing (ms)	0.72	0.81	32.0	16.4	5.64	5.00	37.50

**Table 7:** Classification performance of the extended framework using $CNN_{2}$-lite as subnetworks with triplet pixel features (OA shown in percentage).

	Pines	PaviaU	Salinas
Pair pixel features	95.47	94.51	94.69
Triplet pixel features	**95.91**	**94.79**	**95.98**

**Table 8:** Classification results on the Indian Pines dataset (accuracy shown in percentage).

	SVM	ELM	Pixel- ${\mathrm{CNN}}_{1}$	Patch- ${\mathrm{CNN}}_{9}$	Patch- BsNet	PPF- Framework	SPPF Framework		
							${\mathrm{CNN}}_{2}$	${\mathrm{CNN}}_{2}$-Lite	BsNet
1	78.26	79.40	81.42	84.69	85.83	93.65	92.59	94.22	93.65
2	81.27	85.08	89.81	95.08	87.78	85.08	96.03	97.94	92.54
3	98.59	96.47	95.73	99.29	97.88	96.47	100.0	100.0	100.0
4	98.68	99.06	97.71	99.25	99.06	100.0	99.62	99.43	99.06
5	100.0	100.0	100.0	100.0	100.0	100.0	100.0	100.0	98.92
6	76.94	86.66	88.76	92.88	86.01	89.90	95.34	95.85	95.47
7	65.10	69.84	75.42	81.42	79.82	74.06	90.82	92.20	88.51
8	84.99	89.31	94.12	97.46	94.66	97.20	98.73	98.47	97.46
9	98.78	98.40	97.55	98.78	99.06	99.34	99.81	99.81	99.91
AA(%)	86.96	89.36	91.17	94.32	92.23	92.85	96.99	**97.55**	96.17
OA(%)	80.72	83.80	86.45	90.29	88.49	88.39	95.05	**95.92**	94.96

**Table 9:** Classification results on the Pavia University dataset (accuracy shown in percentage).

	SVM	ELM	Pixel- ${\mathrm{CNN}}_{1}$	Patch- ${\mathrm{CNN}}_{9}$	Patch- BsNet	PPF- Framework	SPPF Framework		
							${\mathrm{CNN}}_{2}$	${\mathrm{CNN}}_{2}$-Lite	BsNet
1	82.69	82.71	86.85	94.44	89.50	87.62	92.38	93.89	93.81
2	87.65	91.23	84.19	86.41	90.19	90.16	90.90	91.71	92.19
3	79.36	79.20	80.75	94.95	88.47	97.53	84.89	83.46	83.10
4	94.24	93.02	93.57	94.34	96.61	99.25	97.14	97.07	97.42
5	99.83	99.30	100.0	99.91	99.83	99.64	99.83	100.0	100.0
6	89.36	91.51	84.54	88.48	94.22	85.62	96.15	95.92	87.49
7	89.38	92.92	92.47	97.26	92.92	73.61	97.35	97.35	97.96
8	81.68	86.79	85.06	90.95	82.25	95.93	86.24	87.71	86.07
9	99.87	99.87	99.73	100.0	99.87	97.93	99.20	99.73	100.0
AA(%)	89.34	90.73	89.68	94.08	92.65	91.92	93.78	**94.09**	93.12
OA(%)	87.25	89.55	86.17	90.17	90.77	86.95	92.09	**92.73**	91.94

**Table 10:** Classification results on the Salinas dataset (accuracy shown in percentage).

	SVM	ELM	Pixel- ${\mathrm{CNN}}_{1}$	Patch- ${\mathrm{CNN}}_{9}$	Patch- BsNet	PPF- Framework	SPPF Framework		
							${\mathrm{CNN}}_{2}$	${\mathrm{CNN}}_{2}$-Lite	BsNet
1	99.00	99.72	99.39	99.72	99.78	99.56	100.0	99.83	99.67
2	99.66	99.55	98.04	99.26	99.86	99.63	99.91	99.72	99.94
3	99.94	100.0	99.55	99.49	99.61	99.72	99.94	100.0	99.55
4	98.74	99.08	99.08	99.41	99.66	99.50	99.66	99.75	99.50
5	98.99	99.27	98.34	97.54	98.75	99.35	99.39	99.64	99.48
6	99.76	99.79	99.55	99.87	99.39	99.57	100.0	100.0	100.0
7	99.50	99.53	99.41	99.67	99.59	99.53	99.94	100.0	99.79
8	70.07	80.87	79.60	82.20	76.73	87.22	87.00	87.55	89.03
9	99.30	99.63	97.70	98.87	99.07	98.52	99.62	99.40	99.47
10	97.66	95.81	91.29	94.54	92.85	96.82	97.95	98.05	98.57
11	99.77	99.42	98.39	98.16	97.24	99.54	99.88	99.88	99.31
12	99.94	100.0	99.36	100.0	99.94	99.88	99.88	100.0	99.88
13	99.58	98.74	98.60	99.86	97.49	99.86	99.44	99.44	99.02
14	98.85	98.28	93.91	97.47	97.82	97.01	99.77	99.66	99.31
15	70.05	76.06	68.81	79.84	75.17	74.65	85.22	86.62	74.31
16	99.63	99.07	98.51	98.13	98.88	99.69	99.56	99.25	99.19
AA(%)	95.65	96.55	94.97	96.50	95.74	96.88	97.95	**98.05**	97.25
OA(%)	88.88	91.99	89.87	92.49	90.62	93.10	94.87	**95.16**	93.99

## Abbreviations

The following abbreviations are used in this manuscript:

HSI	Hyperspectral image
CNN	Convolutional neural network
PPF	Pixel pair feature
SPPF	Spatial pixel pair feature
